# Diagnostic performance of ELISA kits and expanded antigen panels for hemagglutination inhibition assays in pig herds enzootically infected with porcine Influenza A viruses

**DOI:** 10.1186/s40813-025-00464-3

**Published:** 2025-10-27

**Authors:** J. Stadler, K. Grau, K. Lillie-Jaschniski, S. Pesch, A. Graaf-Rau, T. Harder, S. Zoels, R. Fux, M. Ritzmann, M. Eddicks

**Affiliations:** 1 Clinic for Swine at the Centre for Clinical Veterinary Medicine, Ludwig-Maximilians-Universität München, Oberschleissheim, Germany; 2 CEVA Tiergesundheit, Düsseldorf, Germany; 3 Institute of Diagnostic Virology, Friedrich-Loeffler-Institut (FLI), Suedufer 10, 17493 Greifswald-Insel Riems, Germany; 4 Division of Virology, Institute of Infectious Diseases and Zoonoses, Ludwig-Maximilians-Universität München, Oberschleissheim, Germany

**Keywords:** Swine, Influenza A virus, Surveillance, Serological methods, NP-ELISA, HI, Serum samples

## Abstract

**Background:**

A combined approach using both indirect and direct detection methods can improve the diagnostic efficiency of swine Influenza A virus (swIAV) infections. Despite its importance, limited research has been conducted on the diagnostic performance of serological tests commonly used for routine swIAV surveillance. Therefore, the objective of this study was to compare different serological assays, including hemagglutination inhibition (HI) tests using antigen panels of different breadth and three commercially available enzyme-linked immunosorbent assays (ELISAs) to evaluate their suitability as screening tools for identifying herds enzootically infected with swIAV. The diagnostic sensitivity and specificity of the ELISAs were assessed under field conditions, using the HI test as the reference standard.

**Results:**

The HI test panel expanded with various swIAV pandemic strains of regional provenience (HI 3) demonstrated the highest sensitivity (97.77%), while the HI panel including only the four major swIAV subtypes (HI 1) showed the lowest sensitivity (93.74%) and negative predictive value (40.00% compared to HI 4 (incorporating all tested strains)). The level of agreement between the HIs and ELISA tests varied considerably, with the indirect ELISA exhibiting the highest concordance with HI assays. Among the ELISA assays, the indirect ELISA (ELISA 1) achieved the highest sensitivity (95.69%) and overall accuracy (94.26%), albeit with lower specificity (60.00%) when compared with the HI including all strains (HI 4). In contrast, the competitive ELISA (ELISA 2) and the blocking ELISA (ELISA 3) showed lower sensitivity (81.36% and 82.89%) but higher specificity (83.33% and 76.67%, respectively).

**Conclusions:**

This study demonstrates the value of combining different diagnostic tools to improve swIAV surveillance in enzootically infected herds. The indirect ELISA offered high sensitivity and was well suited for broad herd-level screening, while ELISAs based on competitive or blocking formats and HI assays offered higher specificity for confirmatory testing. Among all evaluated methods, the HI assay including locally circulating strains demonstrated the best overall performance and proved useful in detecting additional subtype-level information not identified by PCR. The integration of these approaches enhanced diagnostic precision and supported more effective surveillance strategies, albeit at the cost of increased resource demands.

## Background

 Surveillance for swine Influenza A virus (swIAV) infections is critical due to its public health implications, stemming from the potential for cross-species transmission and, also for reducing economic losses in the pig industry attributable to limited performance of pigs caused by swIAVs clinical impact on pigs’ health [1–4]. The increasing occurrence of enzootically swIAV infected pig herds, often characterized by low intra-herd prevalence (≤ 15%) and non-specific clinical signs in affected animals has further complicated the diagnosis of swIAV [5–9]. Established surveillance approaches for Influenza A in pig herds involve either passive surveillance, triggered by the appearance of clinical signs, or active surveillance in herds without clinical signs [10–16]. Serological tests, including enzyme-linked immunosorbent assays (ELISAs) and the hemagglutination inhibition test (HI) are commonly used to assess the swIAV status of a herd. These tests differ in principle and performance characteristics, ELISAs can either detect antibodies across influenza A subtypes or provide subtype-specific information, while the HI test allows for subtype-specific characterization, depending on the antigen panel used. Since antibodies against swIAV typically develop seven to ten days post infection (p.i.) [17] and can persist for up to six-eight weeks p.i [18]. serology provides a broad time window for detecting infections in unvaccinated pig herds. This is an advantage over the shorter detection window of viral RNA by RT-qPCR in acutely infected pigs [19]. However, the co-circulation of multiple subtypes, and the widespread use of multivalent vaccines in sows often hamper a straightforward interpretation of serological test results [20–22]. Various commercial ELISAs are available, detecting antibodies targeted against the conserved nucleocapsid protein (NP) of IAV such as ID Screen^®^ Influenza A Nucleoprotein Swine indirect (IDvet, Grabels, France), ID Screen^®^ Influenza A Antibody Competition Multispecies (IDvet, Grabels, France), IDEXX Influenza A Ab Test (blocking format, (IDEXX, Maine, United States), BioLisa^®^ kit Influenza A blocking Ab (BioSellal, Dardilly, France) and Influenza virus type A multispecies ELISA (Biovet, Saint-Hyacinthe, Canada) or subtype-specific antibodies against the swIAV HA protein (e.g. Biovet SwineCheck H1N1/H3N2 (Biovet, Saint-Hyacinthe, Canada) [23–27]. The key advantage of NP-ELISA kits is their potential use as a generic serological test, i.e. independent of the IAV subtype which becomes particularly tangible in herds with circulation of multiple swIAV subtypes [23, 27, 28]. This might be of particular importance should incursions of new IAV subtypes of avian origin occur, such as the highly pathogenic strains of subtype H5N1: NP-ELISA kits would still be able to detect a serological response as has been demonstrated in a natural case reported from Italy [29]. Subtype-specific ELISAs, in contrast, have shown low sensitivity (SE) and specificity (SP) in previous studies [24, 26, 27, 30], thereby limiting their diagnostic utility. Serological subtype differentiation therefore still relies on the HI test which is regarded as the gold standard for serological diagnostic [20, 31, 32]. However, HI testing is both, labor- and cost-intensive and is further compromised by antigenic diversity among lineages of the H1 as well as the H3 proteins. Sensitivity of HI tests therefore is an issue also of the degree of antigenic homology between the test HI antigen and the circulating viruses. Due to the possible circulation of multiple subtypes in a single farm, the HI antigen panel needs to be adjusted to obtain reliable data [20, 22, 27, 33–35]. Given the complementary strengths and limitations of HI and ELISA, a combined diagnostic approach may improve surveillance accuracy. While previous studies have compared ELISA and HI assays for the detection of swIAV antibodies, many were conducted prior to the widespread circulation of pandemic H1N1-derived strains or focused only on subsets of pre-classified sera. In addition, several studies relied on experimentally infected animals, which may not reflect the complexity and variability of immune responses under field conditions [24–26, 30, 36, 37]. These approaches may underestimate test limitations related to antigenic diversity or insufficient panel breadth. In light of ongoing viral evolution, the performance of both serological and molecular tests must be regularly re-evaluated to ensure diagnostic accuracy. In this context, our study provides updated and field-relevant insights by systematically comparing three distinct ELISA formats and multiple HI panels—including pandemic and avian strains—using field sera from enzootically infected herds. The broader objective was to provide recommendations for the optimal selection and application of serological tests in routine monitoring programs, particularly in enzootically infected herds with potential circulation of multiple swIAV subtypes and lineages at the same time or consecutively.

## Methods

Blood samples originated from a previous study [7] evaluating novel sampling strategies for the detection of swIAV in 25 enzootically infected sow farms in Germany. Farms were enrolled between March 2021 and February 2022 and included 16 farrow-to-feeder, 8 farrow-to-finish, and 1 breeding herd. Herd sizes ranged from 100 to 7000 sows (median: 475) and were located across various German federal states. Inclusion criteria required on-site nursery facilities, at least ten sows per production stage (farrowing, breeding, gestation), and at least three age groups in the nursery. All farms had a history of swIAV with recurrent respiratory signs (ranging from severe respiratory distress to mild courses characterized by sporadic or transient respiratory signs, such as sneezing or occasional coughing without fever or the need for therapeutic intervention) and prior detection of swIAV by RT-qPCR and/or HI test. In ten farms sows were vaccinated against swIAV using either the trivalent vaccine Respiporc^®^ FLU3 (Ceva, Germany) or the monovalent vaccine Respiporc^®^ FLUpan H1N1 (Ceva, Germany), while in 15 farms no swIAV vaccination was applied. In each farm, blood samples were collected, as part of a diagnostic intervention, from 30 sows. The blood samples were taken from sows (by accessing the jugular vein) in different production stages (gestation, breeding and farrowing) and different parities (gilts, sows parity 2nd -4th, sows >4th parity) and were allowed to clot before centrifugation at 1560 g for 10 min. The serum was recovered, heat-inactivated at 56 °C for 30 min and then frozen at -20 °C at the Clinic for Swine of the LMU Munich until further use. At the Institute of Diagnostic Virology at the Friedrich-Loeffler-Institute, (Greifswald-Insel Riems, Germany) the serum samples were thawed and individually analyzed using three different ELISAs detecting antibodies against the NP of IAV.

The ELISAs investigated consisted of ID Screen^®^ Influenza A Nucleoprotein Swine indirect ELISA (IDvet, Grabels, France, hereafter ELISA 1), ID Screen^®^ Influenza A Antibody Competition Multispecies ELISA (IDvet, Grabels, France, hereafter ELISA 2) and IDEXX^®^ Influenza A Ab test, a multispecies blocking ELISA (IDEXX, Maine, United States, hereafter ELISA 3). The indirect ELISA (ELISA 1) detects anti-influenza A antibodies by immobilizing viral NP antigen on the solid phase and capturing specific antibodies from the serum sample. Detection is achieved through a labeled secondary antibody, which binds to the captured immunoglobulins and generates an amplified signal. The competitive ELISA (ELISA 2) is based on competition between serum antibodies and a labeled monoclonal antibody for a limited number of NP antigen binding sites on the plate. A high antibody concentration in the sample leads to reduced binding of the labeled monoclonal antibody and therefore a lower signal. The blocking ELISA (ELISA 3), often used across species, relies on the principle that specific antibodies in the sample block the binding of a monoclonal antibody to the NP antigen. The more effectively the sample antibodies block this binding, the lower the final signal. This method is robust for multispecies use and helps reduce background noise from non-specific reactions. All ELISAs were performed according to the manufacturer’s instruction. The absorbance was measured at 450nm^c^ (ELISA 1, 2) or 650nm^d^ (ELISA 3) with a microplate reader. The cut-off values for sample to negative (S/N) ratio were interpreted as recommended by the manufacturers: For ELISA 2, samples were considered positive with an S/N% < 50% and negative with an S/N% ≥ 50%. In ELISA 3, a sample was considered positive with an S/*N* < 0.60 and negative with an S/*N* ≥ 0.60. In ELISA 1, samples were considered positive with a sample to positive (S/P) ratio > 0.4 and negative with an S/P ratio ≤ 0.4.

The HI test was performed in accordance with previously published instructions [33, 38]. Prior to implementation the serum samples were treated with a neuraminidase and were adsorbed with chicken erythrocytes to eliminate non-specific agglutination inhibitors or non-specific agglutinins. Afterwards, sera were diluted and incubated with four hemagglutinating units. The final addition of chicken erythrocytes detects the presence or absence of hemagglutination-inhibiting antibodies by the presence or absence of influenza A virus-induced hemagglutination. In the HI tests used, samples were considered positive at a titer level of 1:20 or higher [22].

To assess the benefits of expanding the HI panel, the widely used HI panel (HI 1), which includes four major swIAV subtypes (H1 1C N1, H1 1B N2, H3N2, H1 1A N1), was compared with panels incorporating additional virus subtypes and antigenically distinct virus lineages detected in Germany. The panel HI 2 was expanded with strains from the avian lineages (1C) and the panel HI 3 with strains from the pandemic lineages (1A). Isolates were selected based on technical feasibility (replication in cell culture, stable and sufficient HA titer), epidemiological relevance (local origin, distinct reactivity with field sera), and practical constraints. A detailed description of the strains included in the different HI panels is given in Table 1.

**Table 1:** Composition of HI antigen panels. The table lists swIAV strains grouped by clade and subtype (columns Clade#/Subtype and Strain) and their inclusion in four different HI antigen panels (columns HI panel 1–4). An “x” indicates that the respective strain was included in the corresponding panel. The selected strains represent currently circulating European swIAV isolates of different H1 lineages as well as H3N2. Lineage nomenclature follows Anderson et al. [39]. H1 1A = pandemic lineage 2009; H1 1B = human seasonal lineage 1990s; H1 1C = Eurasian avian-like lineage. Strain designation: A/swine/location/isolate-ID/year

Clade*/ subtype	Strain	HI panel			
		1	2	3	4
H1 1C	A/sw/Haselünne/IDT2617/2003	x	x	x	x
	A/sw/Störmede/IDT13123/2011		x		x
	A/sw/Muldestausee/CEVA26755/2022		x		x
	A/sw/Goch/CEVA26081/2020		x		x
	A/sw/Dülmen/CEVA26060/2019		x		x
	A/sw/Ceradec(F)/24,606/2018		x		x
H1 1C	A/sw/Bernit/CEVA26286/2020		x		x
	A/sw/Lüginghausen/CEVA25404/2018		x		x
	A/sw/Haltern/CEVA253114/2019		x		x
	A/sw/Dietersdorf/CEVA25133/2019		x		x
	A/sw/Nuthe/Urstromtal/26,286/2020		x		x
H1 1B	A/sw/Bakum/1832/2000	x	x	x	x
H1 1A	A/sw/Dessau/AR/2279/2016	x	x	x	x
	A/sw/Hamburg/1580/2009			x	x
	A/sw/WeriteMDCK/IDT22071/2015			x	x
	A/sw/MSV Jena/5258/2009			x	x
	A/sw/Visselhöfede/IDT23245/2016			x	x
H1 1A	A/sw/Papenburg/12,653/2010			x	x
H3N2	A/sw/Bakum/IDT1769/2003	x	x	x	x

*- Nomenclature for strain designation according to Anderson et al. [39]; H1 1A = pandemic lineage 2009, H1 1B = human seasonal lineage 1990s, H1 1C = Eurasian avian-derived

To assess the added value of serology in addition to PCR-based detection, HI titers from sow serum samples were compared with previously published PCR results [7] from the same farms. Briefly, a modified generic Matrix (M)-gene specific Influenza A virus RT-qPCR was performed [40] and positive samples with a Ct-value $\:\le\:$ 32 were forwarded to subtyping RT-qPCR [7]. PCR results were obtained from different age groups (sows, suckling piglets, and nursery pigs), while HI data in the present study were collected exclusively from sows. If the HI titer against a given subtype was at least two titer levels higher than the titer against the subtype detected by multiplex RT-qPCR, it was classified as an additional subtype, in accordance with previous investigations [33].

### Statistical analysis

Statistical calculations were performed using the software IBM SPSS Statistics version 28.0.1.0 for Microsoft^®^ Windows. Cohen’s Kappa analysis was conducted to evaluate the level of agreement between different serological assays used for the detection of swIAV antibodies in swine. Agreement strength was classified according to standard Cohen’s Kappa classification criteria: κ ≤ 0.20 indicates poor agreement, 0.21–0.40 fair, 0.41–0.60 moderate, 0.61–0.80 substantial, and values >0.80 almost perfect [41]. In addition, the study investigated possible associations of sow parity (gilts vs. multiparous sows) and housing localization (farrowing unit, breeding unit, gestation unit) as independent variables with seropositivity (HI 4) by Chi^2^-Test and Odds Ratio. Sensitivity and specificity, positive and negative predictive values as well as accuracy were calculated by the online calculation program MedCalc for Windows, version 19.4 (MedCalc Software, Ostend, Belgium). Level of significance was set at *p* ≤ 0.05.

## Results

### Serological diagnosis of swIAV infection on a herd basis

Antibodies against swIAV were detected in each of 25 herds examined by all serological detection methods, including the three ELISAs and the various HI panels. Out of the 749 blood samples HI 4 revealed positivity to at least one antigen in 722 (96.4%) of the sera, HI 3 in 703/749 (93.9%), HI 2 in 702/749 (93.7%), and HI 1 in 674/749 (90%) of the sera, respectively. In addition, 700 (93.5%) samples were positive by ELISA 1, 603 (80.5%) by ELISA 3 and 590 (78.8%) by ELISA 2.

The indirect ELISA (ELISA 1) showed the highest sensitivity and accuracy among the three ELISAs compared to the HI-based reference standards. However, ELISA 1 exhibited a lower specificity than the other two ELISAs. The lowest sensitivity and accuracy were found for ELISA 2 (Table 2).

**Table 2:** Diagnostic performance of different ELISA tests compared to HI 1 and HI 4 as reference standards

Reference	ELISA test	Sensitivity	Specifity	Predictive Value		Overall Accuracy
				Positive	Negative	
HI 1	1	97.63 (96.17–98.64)	44.00 (32.55–55.94)	94.00 (92.76–95.04)	67.35 (54.40–78.10)	92.26 (90.10–94.07)
	2	84.72 (81.78–87.35)	74.67 (63.30–84.01)	96.78 (95.32–97.80)	35.22 (30.35–40.42)	83.71 (80.87–86.29)
	2*	94.07 (92.01–95.73)	58.67 (46.70–69.92)	95.34 (93.98–96.40)	52.38 (43.53–61.08)	90.52 (88.19–92.52)
	1 + 2	82.79 (79.72–85.56)	76.00 (64.75–85.11)	96.88 (95.39–97.89)	32.95 (28.51–37.71)	82.11 (79.17–84.79)
	3	85.31 (82.41–87.90)	62.67 (50.73–73.57)	95.36 (93.86–96.50)	32.19 (26.95–37.92)	83.04 (80.16–85.66)
HI 4	1	95.69 (93.94–97.05)	60.00 (40.60–77.34)	98.29 (97.37–98.89)	36.73 (26.99–47.70)	94.26 (92.34–95.81)
	2	81.36 (78.32–84.15)	83.33 (65.28–94.36)	99.15 (98.13–99.62)	15.72 (13.01–18.88)	81.44 (78.47–84.16)
	2*	91.38 (89.08–93.32)	73.33 (54.11–87.72)	98.80 (97.84–99.33)	26.19 (20.47–32.85)	90.65 (88.34–92.64)
	1 + 2	79.55 (76.42–82.45)	86.67 (69.28–96.24)	99.31 (98.29–99.72)	15.03 (12.64–17.78)	79.84 (76.78–82.66)
	3	82.89 (79.94–85.58)	76.67 (57.72–90.07)	98.84 (97.80–99.39)	15.75 (12.66–19.43)	82.64 (79.74–85.29)

ELISA 1 (ID Screen^®^ Influenza A Nucleoprotein Swine indirect ELISA), ELISA 2 (ID Screen^®^ Influenza A Antibody Competition Multispecies), and ELISA 3 (IDEXX^®^ Influenza A Ab test) were interpreted using manufacturer-recommended cut-off values, while ELISA 2* was evaluated with adapted cut-offs according to Tse et al. [25]. ELISA test format 1 + 2 represents the combination of tests 1 and 2. HI 1 includes four swIAV subtypes; HI 4 includes all tested strains

The highest agreement among HI tests was observed between the HI 4 and HI 3 tests (κ = 0.779). A substantial agreement was also found among the other HI tests, except for HI 2 vs. HI 3 (κ = 0.622) and HI 4 vs. HI 1 (κ = 0.545) exhibiting moderate agreements. The HI-based assays showed fair agreement with ELISA 2 and ELISA 3 and moderate agreement with ELISA 1, respectively. The highest agreement among ELISA assays was observed between the ELISA 2 and ELISA 3 (κ = 0.632). In contrast, the agreement between the indirect ELISA (ELISA 1) and the competitive or blocking ELISA was only fair (κ = 0.391 ELISA 2; κ = 0.392 ELISA 3), indicating methodological inconsistency between indirect and non-indirect ELISA formats (Table 3).

**Table 3:** Cohen’s kappa coefficient (κ) for agreement between different serological assays for swIAV antibody detection

Serological assay		HI			ELISA		
		1	2	3	1	2	3
HI	4	0.545	0.768	0.779	0.428	0.214	0.211
	1		0.751	0.741	0.492	0.397	0.339
	2			0.622	0.509	0.324	0.320
	3				0.428	0.274	0.244
ELISA	1					0.391	0.392
	2						0.632

Hemagglutination inhibition (HI) assays consist of HI 4 (including all strains), HI 1 (only 4 subtypes), HI 2 (panel expanded with avian strains), and HI 3 (panel expanded with pandemic strains). ELISA assays include ELISA 1 (ID Screen^®^ Influenza A Nucleoprotein Swine indirect ELISA), ELISA 2 (ID Screen^®^ Influenza A Antibody Competition Multispecies) and ELISA 3 (IDEXX^®^ Influenza A Ab test)

The HI 3 provided the highest sensitivity (97.77%) among the evaluated panels, with specificity and PPV remaining at 100%. The NPV (65.22%) was slightly superior to that of HI 2, further reducing the risk of false negatives. In contrast, the HI 1 had the lowest sensitivity (93.74%), NPV (40.00%), and overall accuracy (93.99%) (Table 4).

**Table 4:** Diagnostic performance for detecting SwIAV antibodies of HI 1 (only four subtypes) HI 2 (panel expanded with avian strains) and HI 3 (panel expanded with pandemic strains) compared to HI 4 (including all strains) as reference standard

Reference	Panel	Sensitivity	Specifity	Predictive Value		Overall Accuracy
				Positive	Negative	
HI 4	HI 1	93.74 (91.71–95.40)	100.00 (88.43–100.00)	100.00 (99.45–100.00)	40.00 (33.44–46.94)	93.99 (92.04–95.58)
	HI 2	97.64 (96.24–98.62)	100.00 (88.43–100.00)	100.00 (99.48–100.00)	63.83 (52.45–73.84)	97.73 (96.39–98.67)
	HI 3	97.77 (96.41–98.72)	100.00 (88.43–100.00)	100.00 (99.48–100.00)	65.22 (53.60-75.27)	97.86 (96.55–98.77)

### Influence of localization and parity of the sows on seropositivity by HI

In addition, the study investigated the influence of sow parity (gilts vs. multiparous sows) and housing localization (farrowing unit, breeding unit, gestation unit) on seropositivity using HI positivity (Table 5). Parity was found to be significantly (*p* = 0.002) associated with seropositivity, indicating that multiparous sows were three times more likely to be seropositive compared to gilts.

**Table 5:** Influence of localization and parity of sows on seropositivity by HI

Factor		HI pos. (%)	*p*-value	Odds ratio	95% confidence interval	
Parity	Gilts	38.6	0.002	3.032	1.440	6.387
	≥ 2nd parity	61.4				
Location	gestation	33.7	0.236			
	breeding	33.8				
	farrowing	32.5				

This table presents the effect of sow parity and housing location (gestation, breeding, farrowing unit) on swIAV positivity, as determined by hemagglutination inhibition test

### Concordance of HI serodiagnosis and RT-qPCR based virological diagnosis

swIAV antibodies were detected by at least one of the methods in all 25 farms. In contrast, swIAV RNA had been identified by RT-qPCR in at least one sample from the different specimens (nasal swabs, tracheobronchial swabs, udder skin wipes, environmental wipes, oral fluids) in only 20 of the 25 farms.

In several farms, HI assay results indicated the presence of antibodies against swIAV subtypes that were not detected by subtyping multiplex RT-qPCR. For instance, antibodies against H1 1A N1 were detected in nine farms (36%), although this subtype was not identified by multiplex RT-qPCR on those farms. Similarly, HI responses to H1 1C N1 were observed in five farms (20%), despite the absence of PCR detection (Table 6).

**Table 6:** Overview of vaccination Status, RT-qPCR Results, and HI-based subtype detection across 25 Sow farms

Farm	Vaccination status	RT-qPCR	Hemagglutination inhibition (HI) assay results*
18	Vaccinated	H1 1C N1	
9		H1 1A N2	H1 1C N1
23		H1 1C N1	
21		neg.	Vaccination titres?
11		neg.	Vaccination titres?
7		H1 1C N2, H1 1A N2, H1 1A N2	
16		H1 1C N1	
15		H1 1C N1, H1 1C N2	
14		H1 1C N1, H1 1C N2	
4		H1 1C N2, H1 1C N1, H1 1A N2	
25	Unvaccinated	H1 1C N1	H1 1A N1
24		H1 1C N1	
22		H1 1C N1	
20		neg.	H1 1C N1
19		H1 1C N1	H1 1A N1
17		neg.	H1 1C N1
13		H1 1C N1	H1 1A N1
12		H1 1C N1	H1 1A N1
10		H1 1C N2	H1 1A N1
8		H1 1A N2	H1 1C N1
6		neg.	H1 1C N1, H1 1A N1
5		H1 1C N2	H1 1A N1
3		H1 1C N2, H1 1A N2, H1 1A N2,	
2		H1 1C N2, H1 1C N1, H1 1B N2, H1 1B N1	H1 1A N1
1		H1 1C N2	H1 1A N1

*The HI column presents subtypes under the condition that the HI titer against a specific subtype must exceed the titer against the PCR-detected subtype by at least two titer levels to be classified as an additional subtype. If no additional subtypes are found, the cell remains empty. “Vaccination titers ?” means that it is not possible to clearly differentiate whether the antibodies are a result of vaccination or infection

## Discussion

The continuous evolution of IAV, particularly the recently increased frequency of highly pathogenic avian influenza virus crossing avian-to-mammalian barriers, along with the role of pigs as potential mixing vessels, highlights the need for standardized and robust surveillance strategies for IAV infections in pig herds [1, 2, 42–44]. While ELISA assays offer a rapid, objective, cost-effective, and scalable option for large-scale serological screening, HI assays remain indispensable for detailed characterization at the subtype or even strain level [45, 46]. In this study, we evaluated the performance of three ELISA kits—one indirect, one competitive and one blocking ELISA—together with multiple HI antigen panels. The comparative analysis of the different ELISAs and HI assays revealed significant variation in sensitivity, specificity, and overall accuracy, emphasizing the importance of selecting the appropriate diagnostic method based on the specific surveillance objectives.

Among the evaluated ELISA tests, the indirect ELISA (ELISA 1) demonstrated the highest sensitivity and diagnostic accuracy, making it a strong candidate for initial screening. However, its lower specificity may result in more false positives, and lead to an overestimation of seroprevalence. In contrast, the ELISAs based on a competitive or blocking format—particularly ELISA 2—exhibited higher specificity but lower sensitivity. While their specificity may be advantageous in confirmatory testing, their ability to comprehensively detect seropositive individuals is limited, therefore raising concerns about their value for broad screening in herds with low prevalence. Serial testing with ELISA 1 and ELISA 2 (ELISA with highest specificity), while improving specificity, did not significantly enhance sensitivity, indicating that additional testing with HI tests may still be required for definitive diagnosis. The observed differences between indirect and competitive or blocking formats likely stem from distinct antigen-antibody binding mechanisms. Indirect ELISAs benefits from signal amplification via the use of secondary antibodies, enhancing sensitivity and enabling the detection of even low antibody concentrations. Nevertheless, the method’s broader reactivity potentially compromises specificity [47]. In contrast, competitive and blocking ELISAs aim to improve specificity, either by limiting antibody binding sites (competitive), or by blocking a monoclonal antibody’s access to the antigen (blocking), thereby reducing non-specific interactions. Despite this advantage, these formats often sacrifice sensitivity due to reduced signal amplification and inherent assay competition [46]. The practical implications of these differences are especially relevant in surveillance contexts. Depending on the test system used, herd-level classification may vary considerably—particularly in low-prevalence settings where even minor shifts in sensitivity or specificity can strongly influence prevalence estimates. A more sensitive test may detect more positive samples but include false positives, whereas a more specific assay may underestimate infection rates by missing low-level responses. These discrepancies can impact surveillance data comparability over time and across regions, influence vaccine strategy decisions, and complicate the assessment of true changes in swIAV dynamics. Understanding the diagnostic trade-offs of each assay and applying them according to specific surveillance objectives is therefore essential. Complementary or sequential testing strategies can help balance sensitivity and specificity and improve the interpretability and epidemiological relevance of serological data under field conditions.

Another critical factor influencing test performance is species-specific validation [25]. Some commercially available IAV-specific ELISAs were primarily developed and validated using poultry sera, which may result in suboptimal sensitivity when used for swine. Notably, in our study the indirect ELISA was explicitly validated for swine, whereas ELISA 3 was a multispecies test and ELISA 2 was designed for both swine and avian hosts. This species-specific validation might play a significant role in ensuring the optimal performance of serological assays. In response to findings by Tse et al. [25] who demonstrated that adjusting ELISA cut-off values can improve diagnostic performance on field sera, we re-evaluated ELISA 2 using the optimized S/N cut-off of 0.71 proposed in their study. While this adjustment increased sensitivity, it markedly reduced specificity in our dataset. Notably, Tse et al. [25] only assessed HI-positive sera, whereas we applied the adjusted cut-off to the full range of samples, including those classified as HI-negative. Our findings suggest that cut-off recalibration alone may not sufficiently improve test agreement and must be interpreted cautiously in the context of surveillance goals—whether prioritizing sensitivity or specificity—as well as in light of the test format and the sampled population.

Interestingly, all three ELISA assays showed lower agreement—as reflected by reduced Cohen’s Kappa values (Table 3)—with the HI test panel expanded with pandemic strains compared to the panel expanded with avian strains. This is particularly noteworthy given that the expanded avian strain panel included more recent isolates, as indicated in Table 1, than the expanded pandemic panel. One explanation may be the greater antigenic diversity of pandemic strains due to genetic drift and antigenic changes over time [48, 49], making ELISA-based detection more challenging. Therefore, an update of target antigens used in the ELISAs or a mixture of NP proteins from different lineages might be recommendable to investigate. HI assays are essential for swIAV surveillance because they may allow subtype-level differentiation [31]. This advantage is particularly relevant when distinguishing between co-circulating strains. In addition, Goodell et al. (27) demonstrated that compared to NP-ELISAs, HI assays provide superior specificity and sensitivity, especially during early-stage infection in immunologically naïve animals [26, 50]. Despite its diagnostic advantages, HI testing is labor-intensive, requires standardized antigens, and demands skilled personnel to ensure reproducibility. Additionally, cross-reactivity as well as antigenic distance within and among the H1 protein lineages pose challenges for the HI [27, 37, 51]. This is particularly relevant in farms where multiple subtypes co-circulate or which use vaccination, necessitating adjustments to the diagnostic panel to improve subtype resolution. Furthermore, potential antigenic mismatches with circulating strains can further reduce HI test performance, leading to underestimations of seroprevalence [52]. To address these limitations, we compared a classic HI panel with expanded versions that included pandemic and avian strains. The panel expanded with pandemic strains achieved the highest sensitivity (97.77%) compared to HI 4, (incorporating all strains investigated), thus demonstrating the value of broadening antigenic coverage. In contrast, the classic panel, (including only the four major swIAV lineages), exhibited the lowest sensitivity (93.74%) and negative predictive value (40.00%). Moderate agreement (κ = 0.622) between the two expanded panels suggests that combining both pandemic and avian strains may improve diagnostic breadth. Specifically, the inclusion of an additional strain, such as FLUAV/sw/Muldestausee CEVA26755/2022, which was primarily responsible for the positive results in the expanded avian panel, along with antigenically distinct pandemic strains, enhances panel effectiveness. These findings support the use of comprehensive HI panels encompassing both historically significant and newly emerging strains to enhance surveillance efficacy. However, it should be noted that expanding antigen panels is associated with increased costs and logistical complexity, which may limit their routine applicability. Given the inherent limitations of individual assays, a combined approach, utilizing both ELISA and HI, provides a more robust and comprehensive strategy for swIAV surveillance.

This study has several limitations that should be acknowledged. First, the true infection status of the sampled animals was unknown, as the sera originated from a previous field study with no defined gold standard; the HI assay was therefore used as reference method despite its known limitations such as variability in standardization and dependency on antigen selection. However, in the absence of a true gold standard in field settings, the HI assay was chosen as a practical and justified benchmark due to its established role in surveillance programs—especially as we implemented broadened antigen panels tailored to regionally circulating strains. Furthermore, the interpretation of serological results was complicated by the co-circulation of multiple subtypes and the use of vaccines in some herds. In vaccinated herds, Furthermore, the interpretation of serological results was complicated by the co-circulation of multiple subtypes and the use of vaccines in some herds, as vaccinated- and infection-induced antibodies cannot be distinguished. Lastly, although the HI panels were expanded to include locally circulating strains, this approach may limit the applicability of our findings to other regions with different subtype distributions. Taken together, these factors should be considered when interpreting the results and designing future surveillance programs. Nevertheless, despite these limitations, the study provides valuable insights into the comparative performance of current serological assays and highlights key considerations for optimizing swIAV surveillance strategies under field conditions.

Beyond assay performance, we investigated the impact of sow parity (gilts vs. multiparous sows) and housing location (farrowing unit, breeding unit, gestation unit) on seropositivity (Table 5). Multiparous sows had significantly higher odds of testing seropositive (OR = 3.032, *p* = 0.002) compared to gilts, suggesting cumulative exposure across reproductive cycles. In contrast, housing location showed no significant effect (*p* = 0.236), indicating consistent viral circulation across units, possibly due to shared airspace or personnel movements.

Our findings, in accordance with previous studies [33], emphasize the complementary use of serology and PCR in surveillance programs. RT-qPCR allows the identification of currently circulating viruses and enables subtyping based on viral RNA. However, it is limited by the short diagnostic window and influenced by sampling timing and the shedding status of individual animals. In contrast, serological methods—particularly the HI assay—capture retrospective exposure and may uncover subtypes that circulate at low prevalence or escape PCR detection. Indeed, in five out of 25 farms, (data presented elsewhere (Stadler et al. [7]) RT-qPCR failed to detect swIAV, while serology confirmed prior exposure, however, in two farms it remains unclear whether the detected antibodies resulted from natural infection or vaccination. Additionally, comparison of HI titers with previously obtained PCR data from the same farms revealed antibody responses against subtypes that were not detectable by PCR. Interestingly, hints towards the potential circulation of additional subtypes and lineages were more frequently detected in unvaccinated farms compared to vaccinated ones (Table 6). This may reflect broader antigenic circulation in immunologically naïve herds or the presence of subtype-specific vaccine-induced antibodies that may mask natural infections serologically. While this observation suggests the value of vaccination in reducing viral diversity and circulation, it also underscores the challenge of interpreting serological data in vaccinated populations. In particular, effects related to original antigenic sin and cross-boostering between different subtypes and lineages may complicate the interpretation of results [53, 54].

These findings are consistent with experimental co-infection studies in pigs, which suggest that vaccination may limit within-host reassortment and reduce the emergence of novel IAV genotypes [55]. However, field conditions are more complex, and subclinical infections or undetected co-infections may still lead to the silent spread of antigenically diverse viruses. Taken together, our data demonstrate that the integration of serological and molecular tools improves the diagnostic depth of swIAV surveillance and supports more effective control strategies. This combined approach is particularly critical in partially vaccinated herds with ongoing circulation of diverse virus populations, where clinical signs may be subtle and antigenic drift necessitates the regular reassessment of diagnostic tools.

## Conclusions

The findings of this study highlight the necessity of a multi-tiered diagnostic strategy for effective swIAV surveillance in enzootically infected herds. Given its high sensitivity, the indirect ELISA serves as the most suitable tool for broad screening, particularly in herds with low prevalence (≤ 15%). However, due to its low specificity, a second test with higher specificity, such as a competitive ELISA or HI assay, is essential for confirmatory testing. The HI panel expanded with local strains demonstrated the best overall performance, highlighting the importance of tailoring antigen panels to regional epidemiological situations. Furthermore, the HI—can uncover circulating subtypes that may go undetected by molecular assays, as demonstrated by the serological identification of pandemic strains in PCR-negative herds.

Importantly, the diagnostic value of serological assays depends on up-to-date antigenic coverage, which in turn requires ongoing virological surveillance. Finally, the integration of molecular and serological methods—each with its distinct strengths— enhances the understanding of swIAV dynamics and enables the detection of a broader range of subtypes, thus leading to more effective control strategies.

## Data Availability

No datasets were generated or analysed during the current study.

## Abbreviations

Ct: Cycle threshold
ELISA: Enzym-linked-immunosorbent-assay
ELISA 1: ID Screen^®^ Influenza A Nucleoprotein Swine indirect ELISA (IDvet, Grabels, France)
ELISA 2: ID Screen^®^ Influenza A Antibody Competition Multispecies ELISA (IDvet, Grabels, France)
ELISA 3: IDEXX^®^ Influenza A Ab test (IDEXX, Maine, United States)
FLI: Friedrich-Loeffler-Institute
HI: Hemagglutinin inhibition
HI 1: HI panel with four swIAV lineages (H1 1C N1,H1 1B N2,H3N2,H1 1A N1)
HI 2: HI panel expanded with avian strains
HI 3: HI panel expanded with pandemic strains
HI 4: HI panel including all strains
IAV: Influenza A virus
LMU: Ludwig-Maximilians-Universität
NPV: Negative predictive value
NP: Nucleoprotein
OF: Oral fluid(s)
PPV: Positive predictive value
p.i: Post infection
RT-qPCR: Real-time quantitative PCR
swIAV: Swine Influenza A virus
S/N: Sample to negative ratio
S/P: Sample to positive ratio
Se: Sensitivity
Sp: Specificity
